# Gut microbiota and atherosclerosis: role of B cell for atherosclerosis focusing on the gut-immune-B2 cell axis

**DOI:** 10.1007/s00109-020-01936-5

**Published:** 2020-07-31

**Authors:** Lin Chen, Tomoaki Ishigami, Hiroshi Doi, Kentaro Arakawa, Kouichi Tamura

**Affiliations:** 1 Department of Medical Science and Cardio-Renal Medicine, Graduate School of Medicine, Yokohama City University, 3-9, Fukuura, Kanazawa-ku, Yokohama, Kanagawa Japan; 2 Department of Cardiology, Sir Run Run Hospital, Nanjing Medical University, Long Mian Avenue 109 Jiangning, Nanjing, Jiangsu China

**Keywords:** Commensal microbiota, Atherosclerosis, Inflammation, B2 cells

## Abstract

Atherosclerosis is the leading cause of cardiovascular mortality and morbidity worldwide and is described as a complex disease involving several different cell types and their molecular products. Recent studies have revealed that atherosclerosis arises from a systemic inflammatory process, including the accumulation and activities of various immune cells. However, the immune system is a complicated network made up of many cell types, hundreds of bioactive cytokines, and millions of different antigens, making it challenging to readily define the associated mechanism of atherosclerosis. Nevertheless, we previously reported a potential persistent inflammatory process underlying atherosclerosis development, centered on a pathological humoral immune response between commensal microbes and activated subpopulations of substantial B cells in the vicinity of the arterial adventitia. Accumulating evidence has indicated the importance of gut microbiota in atherosclerosis development. Commensal microbiota are considered important regulators of immunity and metabolism and also to be possible antigenic sources for atherosclerosis development. However, the interplay between gut microbiota and metabolism with regard to the modulation of atherosclerosis-associated immune responses remains poorly understood. Here, we review the mechanisms by which the gut microbiota may influence atherogenesis, with particular focus on humoral immunity and B cells, especially the gut-immune-B2 cell axis.

**Graphical abstract:**
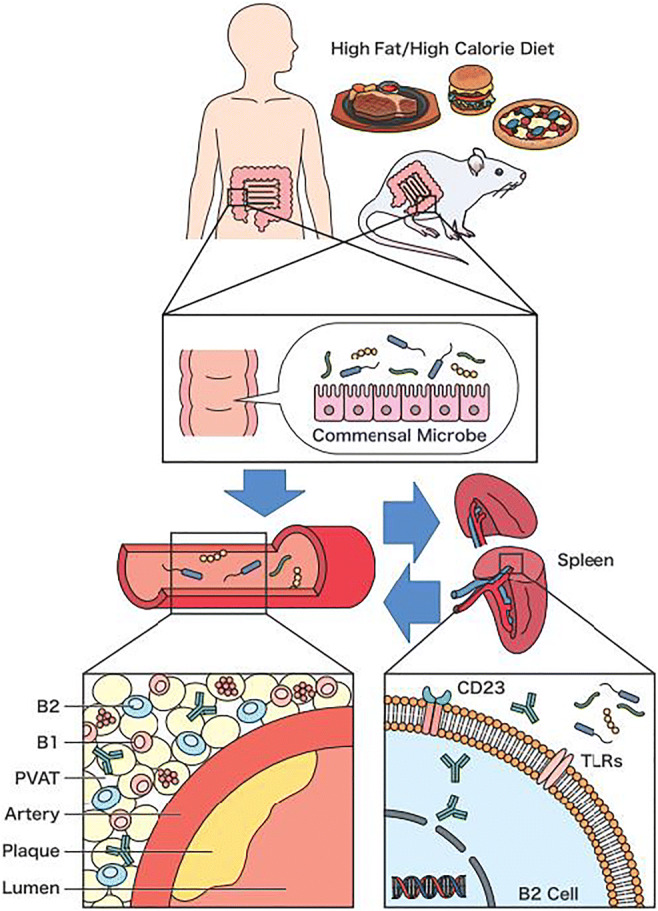
Under high-fat and high-calorie conditions, signals driven by the intestinal microbiota via the TLR signaling pathway cause B2 cells in the spleen to become functionally active and activated B2 cells then modify responses such as antibody production (generation of active antibodies IgG and IgG3), thereby contributing to the development of atherosclerosis. On the other hand, intestinal microbiota also resulted in recruitment and ectopic activation of B2 cells via the TLR signaling pathway in perivascular adipose tissue (PVAT), and, subsequently, an increase in circulating IgG and IgG3 led to the enhanced disease development. This is a potential link between microbiota alterations and B cells in the context of atherosclerosis.

Under high-fat and high-calorie conditions, signals driven by the intestinal microbiota via the TLR signaling pathway cause B2 cells in the spleen to become functionally active and activated B2 cells then modify responses such as antibody production (generation of active antibodies IgG and IgG3), thereby contributing to the development of atherosclerosis. On the other hand, intestinal microbiota also resulted in recruitment and ectopic activation of B2 cells via the TLR signaling pathway in perivascular adipose tissue (PVAT), and, subsequently, an increase in circulating IgG and IgG3 led to the enhanced disease development. This is a potential link between microbiota alterations and B cells in the context of atherosclerosis.

## Introduction

Atherosclerotic diseases comprise systemic disorders that represent a leading cause of mortality and morbidity worldwide. Although the molecular mechanisms responsible for the development of atherosclerosis are not completely understood, studies over the past decade have highlighted the critical role of the immune system in this process. In particular, cells, both the innate (macrophages) and adaptive (T cell and B lymphocytes) branches, of the immune system appear to play an important role in the development of this common condition [1–3]. In addition, recent studies have revealed that the gut microbiome exerts direct effects on the immune responses that regulate chronic inflammatory diseases including rheumatoid arthritis, inflammatory bowel disease, and atherosclerosis [4, 5]. Moreover, abnormal cholesterol concentrations, an unhealthy diet, and alterations in the gut microbiota have been linked to atherosclerosis progression [4]. Accordingly, the intriguing relationship between commensal microbes and atherosclerosis has received increasing attention over the past few years. However, the specific mechanisms whereby commensal microbes regulate the development of atherosclerosis are just beginning to be elucidated [6, 7], with the role of the immune system in commensal microbe-derived atherosclerosis, i.e., metabolism-independent pathways, remaining largely unexplored. Therefore, the purpose of this review is to highlight current knowledge regarding the complex interplay between the microbiota and atherosclerosis via the immune system, with a particular focus on the associated roles played by humoral immunity, including both B1 and B2 cells.

## Association of commensal microbiota with cardiovascular diseases

Atherosclerosis constitutes the main contributor to cardiovascular mortality, which is strongly associated with risk factors such as gender, age, genetic background, unhealthy diet, smoking, hypertension, diabetes mellitus, obesity, hyperlipidemia, and socioeconomic deprivation [8, 9]. However, minimizing such risk factors does not altogether protect against atherosclerosis. At least a 50% residual risk may remain, even in conjunction with high-potency statin therapy [10, 11]. This is because inflammation and hypercholesterolemia comprise the two key etiological factors for atherosclerosis [2, 12], whereas current therapeutic options for treating or preventing atherosclerosis mainly focus on lipid control alone, rather than resolving inflammation [13].

Bacterial infection has been proposed as a trigger of inflammation in atherosclerosis [13, 14]. To date, most epidemiological evidence has supported that a relationship between infection and atherosclerosis exists, based on associations between circulating antibacterial antibodies and atherosclerosis. For example, foreign antigenic stimuli, such as *Porphyromonas gingivalis*, *Chlamydia pneumoniae*, enterovirus, and cytomegalovirus, have been identified as potentially causative or bystander participants [15]. Furthermore, several studies have found that *C. pneumoniae* is present in the atherosclerotic lesions of patients with previous exposure and that infection with this bacterium may exacerbate atherosclerosis in animals [14, 16]. In addition to *C. pneumoniae*, almost 50 bacterial species have been detected in atherosclerotic plaques, whereas none was found in control tissues [5, 17].

Moreover, bacteria-host interactions have been associated with the initiation, perpetuation, and re-exacerbation of atherosclerotic lesions, eventually leading to thrombus formation and acute coronary syndromes or stroke [18–22]. Recent studies also showed that atheromas collect bacteria from the circulation and microbial molecular signatures have been detected at progressively higher frequencies in advanced lesions [7, 23]. In addition, some studies showed a correlation between aortic stiffness and blood level of soluble CD14, the main endotoxin receptor and defense against gram-negative bacteria, with high levels resulting in aortic stiffness [24, 25].

Although such evidence supports that bacterial infection may play a role in the atherosclerotic process from initial endothelial dysfunction to clinical manifestations, whether an infection initiates or augments atherosclerosis development remains uncertain. For example, only a few infectious agents such as *Aggregatibacter actinomycetemcomitans*, *C. pneumoniae*, *Helicobacter pylori*, and *P. gingivalis* have been shown to potentially contribute to atherosclerosis by increasing lesion areas in animal models [26]. Furthermore, several large, randomized clinical trials involving antibiotic therapy have shown no benefit, to date, regarding cardiovascular endpoints [27], with the recent suggestion that the organization of bacteria in antibiotic-resistant biofilms may have contributed to these negative results [28, 29]. This reflects the view that the total pathogenic infectious burden in any individual may be more important than any singular microbe as a risk factor for cardiovascular disease [30]. Therefore, an important endogenous bacterial source of infection, the ability of commensal microbes to potentially exert a substantial impact on atherosclerosis, has been recognized [7, 31].

Consistent with this observation, numerous studies have reported the detection of bacterial DNA in atherosclerotic lesions, as well as in human atherosclerotic plaques [7, 17, 32]. In particular, pyrosequencing results revealed that the bacteria in lesions are derived from the gut and oral cavity [7], suggesting the possible involvement of oral and gut microbiota in the development of the disease.

Furthermore, fecal samples from healthy individuals and patients with symptomatic atherosclerosis were found to differ by several species. For example, the genus *Collinsella* was enriched in patients with symptomatic atherosclerosis, whereas *Eubacterium* and *Roseburia* were enriched in healthy controls [33]. Moreover, in patients with coronary artery disease, the number of *Lactobacillales* and the ratio of *Firmicutes* to *Bacteroidetes* increased, along with the levels of *Escherichia coli*, *Klebsiella* spp., *Enterobacter aerogenes*, *Ruminococcus gnavus*, *Eggerthella lenta*, *Streptococcus* spp*.*, *Lactobacillus salivarius*, *Solobacterium moorei*, and *Atopobium parvulum*. In comparison, the number of Bacteroidetes, *Roseburia intestinalis*, *Faecalibacterium prausnitzii*, *Bacteroides* spp*.*, *Prevotella copri*, and *Alistipes shahii* decreased [34, 35].

Accordingly, treatment with systemic antibiotics in humans showed no reduction in cardiovascular event rates, possibly owing to the specific eradication of gram-positive strains by azithromycin, whereas gram-negative (LPS-containing) intestinal bacteria remained unaffected [28]. Moreover, the approach of using broad-spectrum antibiotics to deplete the gut microbial population also causes a reduction in numerous beneficial products derived from commensal microbes [6, 36].

## Gut dysbiosis and inflammation in atherosclerosis

The specific mechanisms whereby commensal microbes may regulate the development of atherosclerosis are just beginning to be elucidated. However, numerous studies have identified the ability of commensal microbe-derived metabolites to act as hormones or bioactive metabolites modulating cardiovascular disease risk. These have focused on metabolism-dependent mechanisms, including the gut microbe-derived trimethylamine N-oxide (TMAO) pathway [37], the short-chain fatty acids (SCFA) pathway, and the primary and secondary bile acids pathways. In contrast, metabolism-independent pathways, particularly the role of the immune system in commensal microbe-derived atherosclerosis, remain largely unexplored.

It is considered possible that the intestinal microbiota can regulate atherosclerosis development via bacterial wall compounds, such as endotoxin and lipopolysaccharide (LPS), or indirectly through the regulation of innate immunity and chronic inflammatory tone by bacterial products [38]. For example, in a 5-year epidemiological study of 516 middle-aged men and women, those with plasma LPS levels over 50 pg/mL exhibited a threefold increased risk of developing atherosclerosis, whereas the subpopulation of smokers or ex-smokers with the same LPS level evinced a 13-fold increase [39]. Nevertheless, to our knowledge, intervention studies to lower LPS plasma levels and thereby subsequently decrease cardiovascular disease risk have not been conducted, even though such results would verify the importance of LPS in the etiology of cardiovascular disease [39]. Furthermore, microRNA components of intestinal microbiota [40] can affect macrophage function and subsequent inflammatory tone. In addition, lack of microbiota reduced plasma LPS levels, along with pro-inflammatory cytokine gene expression in macrophages and the aorta, during atherosclerosis development [41].

The interaction of microbes, as well as the components of the bacterial cell, with the immune system was previously considered to be most active in the distal gut [42, 43]. However, recent studies have found that low levels of microbiota can also enter the bloodstream to cause chronic low-grade inflammation systemically [6, 44]. The phenomenon whereby low level of gut-derived bacteria can appear in the circulation is commonly referred to as “metabolic endotoxaemia” and it has been found to be prevalent in atherosclerosis [44].

Generally, the gut barrier plays a critical role in preventing the translocation of bacterial components. This barrier is efficient when the microbiota is complex and stable, whereas under some conditions, such as diets high in fat and cholesterol or certain diseases, major changes could be induced in the host microbiota composition, which in turn has been associated with increased intestinal permeability [45–47]. For example, we and others have found that mice fed a high-fat diet exhibited increased intestinal permeability and decreased expression of genes encoding tight junction proteins, including zonula occludens-1 (ZO-1), claudin-1, and occludin, whereas the administration of antibiotics in conjunction with the high-fat diet effectively ameliorated these negative effects [48, 49]. In follow-up experiments, it was confirmed that the obese mice exhibited the highest levels of intestinal permeability; moreover, obesity-prone rats were also found to exhibit increased gut permeability, plasma LPS, and inflammation, albeit with reduced epithelial barrier function as compared with obesity-resistant rats [50–52]. In addition, our group also found that lubiprostone attenuates the development of atherosclerotic lesions by ameliorating leaky gut syndrome-induced inflammation through the restoration of the intestinal barrier [53]. Consistent with these observations, individuals with inflammatory bowel disease were at higher risk of developing coronary artery disease, despite having lower rates of traditional risk factors than their age-matched controls, in a longitudinal cohort study [39]. Targeted sampling studies have shown that LPS levels are higher in blood samples recovered from the hepatic vein as opposed to the systemic circulation (direct sampling from the ventricles), providing direct evidence that LPS can be translocated from the gut [54]. Therefore, owing to the compromise of the intestinal barrier, commensal microbes or commensal microbe-derived molecules, such as LPS or peptidoglycan, can readily enter the bloodstream and exert systemic effects, including the induction of infection or chronic low-grade inflammation and immunoreaction, affecting multiple immune populations.

Furthermore, it has become clear that microbiota-derived bioactive compounds can signal to distant organs, contributing to the development of cardiovascular disease states [55]. For example, outer membrane components of gut microbiota such as LPS, other virulence factors, and pathogen-associated molecular patterns (PAMPs) can be detected in human tissues and trigger local and systemic inflammatory responses [56–58]. In particular, increased intestinal microbiota-derived LPS load from the colon lumen was shown to be associated with various metabolic abnormalities, including the induction of adipose inflammation. In addition, LPS-induced inflammatory cytokines in perivascular adipose tissue (PVAT), which surrounds nearly all blood vessels, can act in a paracrine manner to exacerbate vascular inflammation and atherosclerosis. Similarly, through the circulation, bacteria can reach visceral fat or atheromas, directly promoting local inflammatory cascades or eliciting a specific immune response [59, 60], thereby indirectly influencing host metabolism and systemic inflammation [15, 61].

## Commensal microbe–induced atherosclerosis via immune response

It has long been understood that our immune system can sense various types of bacterial components, such as LPS and peptidoglycan, via cognate pattern receptors located on immune cells [62], and then activate several inflammatory pathways. In general, these pathways involve Toll-like receptors (TLRs) and nucleotide oligomerization domain (NOD)–like receptors (NLRs) [63]. In particular, circulating LPS, derived from different gut microbial species, are believed to confer their deleterious effects on developing atherosclerosis primarily through the TLRs and their receptors, for example, cluster of differentiation 14 (CD14), with TLR receptor activation downstream signaling cascades including nuclear factor kappa B (NF-κB) and c-Jun N-terminal kinase pathways. Activation of the NF-κB pathway promotes gene expression that recruits and activates inflammatory cells and downstream molecules such as cytokines, including the pro-inflammatory factors interleukin-6 (IL-6), IL-1, IL-27, tumor necrosis factor alpha (TNF-α), inducible nitric oxide synthase, and leukocyte adhesion molecules [64]. Similarly, activation of the c-Jun N-terminal kinase pathway leads to the upregulation of stress response genes and is implicated in pathological cardiac events [54].

For example, through the use of TLR and low-density lipoprotein (LDL) receptor double knockout mice, several studies have demonstrated that TLRs may be contributors to atherosclerosis development [65]. Indeed, Ding et al. [66] found that a TLR deficiency reduced atherosclerosis without any effect on inflammation. Moreover, inactivation of the TLR pathway by deletion of TLRs or the downstream cytosolic adaptor, myeloid differentiation factor-88 (Myd88), reduces aortic lesions in apolipoprotein E–deficient (ApoE−/−) and LDL receptor–deficient (Ldlr−/−) mice [67]. Notably, all these models showed a reduction of lesion area and regional lipid content without any significant alteration of plasma cholesterol levels. Consistent with these findings, clinical investigations have revealed that the upregulation of TLRs was associated with inflammatory activation in human atherosclerosis and promoted the development of atherosclerosis [68–70].

Additionally, another gut microbial PAMP, peptidoglycan, was also found to be associated with atherosclerosis via NLRs. Through peptidoglycan recognition, NLRs promote intracellular bacteria clearance through a program involving NF-κB and mitogen-activated protein kinase (MAPK) signaling pathways [71]. Recently, knockout of NOD1 in mice was shown to significantly reduce the development of atherosclerotic lesions [63]. Moreover, some NOD2-knockout mouse studies revealed that NOD2 represents a critical regulator of intestinal bacterial immunity and helps to maintain the integrity of the gut barrier [72]. In addition, other PAMPs have been identified that may promote atherosclerosis development through NLR protein 3 (NLRP3)-inflammasome-caspase-dependent signaling pathways, causing the conversion of pro-IL-1 beta and pro-IL-18 into active cytokines and subsequent induction of inflammation [73–76].

Through these pathways, the microbiota activate the innate and adaptive immunity via receptors on endothelial cells, innate lymphoid cells (ILCs), dendritic cells (DCs), other myeloid cells, and lymphocytes [77–79]. In turn, this provides stimuli for the activation of leukocytes and arterial cells within atheromas [80].

Moreover, microbial antigens are also associated with the molecular mechanism termed “molecular mimicry” [81], as self-peptides such as heat shock proteins (e.g., mycobacteria, Chlamydia) have also been found to be associated with atherosclerosis [82]. For example, Binder et al. showed that pneumococcal vaccination decreases atherosclerotic lesion formation through a molecular mimicry mechanism between *Streptococcus pneumoniae* and oxidized LDL [83]. In addition, a recent study has reported that auto-antibodies produced by B lymphocytes are present in plaques, which may cross-react with outer membrane proteins of bacteria, as well as with a cytoskeleton protein involved in atherogenesis [60]. These findings demonstrated that, in human atherosclerotic plaques, a local cross-reactive immune response may occur, wherein antibodies cross-react with a bacterial antigen and a self-protein. These results also illustrated that antibodies and B lymphocytes could play an important role in the disease process [60, 84].

## Crosstalk between microbiota and B cells

As an important component of the immune system, B cells play a critical role in inflammation through their ability to detect and process antigens, terminally differentiate into plasma cells, and produce antibodies or cytokines [85]. B cells can also affect atherosclerosis development via production of atherogenic antibodies [86] and secretion of pro-inflammatory cytokines, including TNFα, which represent T cell–independent pathways [87]. However, in contrast to the ability of macrophages and specific T cell subsets to promote inflammation in the vessel wall during atherosclerosis [88–90], B cells may have a more complex role in atherosclerosis development through antibody production, which is not yet fully elucidated [91]. For instance, B1 cell–derived natural IgM antibodies have consistently been shown to be athero-protective [92, 93], while B2 cell responses may promote atherogenesis by supporting pro-atherogenic T cells [94].

In general, commensal microbes or commensal microbe-derived LPS or peptidoglycan can be selectively recognized by the hosts’ innate immune TLRs in B cells [95–97]. Similar to other immune cells, B cells also exhibit variations in TLR expression patterns; specifically, the signaling via MyD88 is able to modify B cell responses, such as antibody production, antigen presentation, and cytokine secretion [98–100]. Notably, recent studies have demonstrated that activation of TLRs, via MyD88 signaling in B cells, is necessary for antibody responses to T cell–dependent (TD) antigens and to influence B cell tolerance, which leads to pathogenic autoantibody production [96, 97, 100]. However, the role of the TLR signaling pathway in B2 cells during atherosclerosis development is not fully elucidated. Nevertheless, in our previous study, we found that under hyperlipidemic conditions, signals driven by the microbiota via the TLR signaling pathway may cause B2 cells to become functionally active, potentially leading to the generation of active antibodies, cytokines, and chemokines, thereby providing a mechanism in which they may be contributing to atherosclerosis development [1, 8, 53].

Extensive studies of the peripheral blood, peritoneal cavity, and other lymphoid tissues have identified B1 and B2 cells as the two main B cell subsets in mice, based on their developmental origin [101]. In addition, the B2 cells that represent the vast majority of B cells, including follicular (FO) as well as marginal zone (MZ) B cells [102] respond to antigen presentation in a TD manner undergoing proliferation, affinity maturation, and isotype class switching to produce a large amount of highly specific antibodies against foreign pathogens [102].

B2 cells reside mainly in the spleen, accounting for 60% of the total number of splenic lymphocytes. Splenic B2 cells comprise approximately 80% FO B2 cells and 10% MZ B2 cells [102]. In general, FO B2 cells predominantly participate in TD antibody responses to highly specific determinants that are usually associated with microbial proteins [103]. In contrast, as MZ B2 cells are peripheral to the FO B2 cells and reside in the marginal sinuses of the spleen [104], the location of the interface between the spleen and the circulation, they are thus located at the first line of defense against blood-borne antigens [103, 105]. MZ B2 cells predominantly give rise to rapid T cell–independent (TI) antibody responses to highly conserved carbohydrate and glycolipid determinants associated with microbes, producing TI antibodies such as the IgM response that bridges the gap between infection and the production of TD antibodies [106–114]. Moreover, the high expression of MZ B2 cells of antigen-presenting CD1d molecules, which bond lipids and glycolipids, allows them to act as antigen processing cells for the activation of natural killer T cells (NKT cells) [115].

In parallel, commensal microbes stimulate the homing of DCs, along with neutrophils, to the MZ of the spleen, which has an important role in the activation of MZ B2 cells [107]. In addition, recent findings show that neutrophils occupy peri-MZ areas of the spleen in the absence of infection, being recruited via a non-inflammatory pathway that originates during fetal life and accelerates after birth, a time that coincides with the colonization of mucosal surfaces by bacteria [114]. Moreover, serum natural IgG levels are severely reduced, whereas serum natural IgM levels are normal in germ-free animals [116]. These results indicate the essential role of commensal microbes in the activation of MZ B2 cells and B2 cell–mediated IgG antibody production. Additionally, unique roles were reported for MZ B2 cells in atherosclerosis development, involving enhanced pro-atherogenic T cell responses in mice [117].

Intestinal commensal microbes have come to be accepted as an important antigen source for the activation of specific splenic B2 cells in association with arteriosclerosis. The main pathways by which host and commensal microbiota interact are when commensal microbiota or their metabolites enter the hosts’ circulation. As with other infection antigens, the innate immune system is capable of sensing various types of commensal microbiota components via TLRs, which then signal via MyD88-dependent pathways to activate NF-κB-driven pro-inflammatory signaling, subsequently leading to an adaptive immune response [100, 118]. For example, the results of our recent study collectively demonstrated that under hyperlipidemic conditions, signals driven by the intestinal microbiota, via the TLR signaling pathway, cause B2 cells in the spleen to become functionally active. Subsequently, the activated B2 cells then modify responses such as antigen presentation and antibody production, thereby potentially contributing to the development of atherosclerosis [1].

In addition, Hamze et al. [119] used laser capture microdissection to analyze individual lymphocytes in dissected coronary arteries, finding that the majority of B cells were present in the adventitia of these arteries and that they primarily expressed markers associated with the activated plasmoblast phenotype, suggesting the cells were active at the sites of disease. Moreover, the presence of B cells in the aortic adventitia has been supported by considerable evidence in human and murine models of atherosclerosis, configuring artery tertiary lymphoid organs (ATLO) in blood vessels [120, 121]. Recent studies further support that B cell activation in the adventitia is important for regulating atherosclerosis [119, 122]. In comparison, the PVAT, which is intimately associated with the adventitial layer of the vessel wall, has been implicated, through paracrine effects on the vasculature, to play a pivotal role in the pathogenesis of atherosclerosis [123]. PVAT constitutes a complex mixture of various cell types including immune cells, such as macrophages, T cells, and B cells [124, 125] that histologically form fat-associated lymphoid clusters (FALCs) [126]. Due to the close interaction between PVAT and adventitia, immune cells in the PVAT likely contribute to the development of atherosclerosis. Consistent with this conjecture, we have demonstrated that, under hyperlipidemic conditions, intestinal microbiota may enter the blood owing to the reduced intestinal mucosal barrier capacity. This may then result in the recruitment and ectopic activation of B2 cells, especially FO B2 cells, via the TLR signaling pathway in PVAT, and, subsequently, in an increase in circulating IgG and IgG3, ultimately leading to enhanced disease development [1].

## Conclusions and outstanding questions

New insights regarding how atherosclerosis can be affected by commensal microbiota have been provided over the past decade; however, the underlying molecular and cellular mechanisms remain largely unexplored. In this review, we have provided an overview of the metabolism-independent pathways in atherosclerosis development and discussed the possible mechanisms involving splenic B2-B cell activation following commensal microbe dysbiosis and translocation into the bloodstream. As inflammation constitutes a key etiological factor for atherosclerosis, future research must seek to pinpoint the specific immune response mediated by microbiota. In particular, the impact of the microbiota on immune cells and its consequences for atherogenesis await further elucidation.

Finally, various studies have revealed the roles of athero-protective B1 cells and athero-promoting B2 cells, and the disruption of the balance between B1 cells and B2 cells may lead to the progression of atherosclerosis. Therefore, targeting activated B2 cells or induced athero-protective B1 cells might be one of the therapeutic procedures for the subjects with atherosclerosis. Furthermore, a potential translational extension of the current research would be to better characterize the specific humoral immunity in individuals with atherosclerosis. We sought to confirm the existence of antibodies specific for antigens derived against commensal microbes and to develop in vitro diagnostic procedures for assessing the current immunological status of atherosclerosis patients. For instance, we found an IgG3 class of antibodies specific for bacterial antigens in these patients that may prove useful as a translation tool in clinical settings (unpublished observation). Also of clinical relevance, probiotics, and not antibiotics, may effectively alter the state of dysbiosis in subjects with atherosclerosis, while also reducing the specific effect of commensal microbes in the development of atherosclerosis. Additionally, interventional approaches can also be applied to enhance the intestinal function of subjects with atherosclerosis. For instance, patients with coronary heart disease and constipation might present interventional opportunities involving the use of laxative agents for improving intestinal commensal microbiota [53, 127, 128].
